# Wearable Technologies for Healthy Ageing: Prospects, Challenges, and Ethical Considerations

**DOI:** 10.14283/jfa.2024.19

**Published:** 2024-02-21

**Authors:** S. Canali, A. Ferretti, V. Schiaffonati, Alessandro Blasimme

**Affiliations:** 1 Politecnico di Milano, Milan, Italy; 2 Eidgenossische Technische Hochschule, ETH Zürich, Zurich, Switzerland

**Keywords:** Wearables, healthy aging, intrinsic capacity, ethics, digital health

## Abstract

Digital technologies hold promise to modernize healthcare. Such opportunity should be leveraged also to address the needs of rapidly ageing populations. Against this backdrop, this paper examines the use of wearable devices for promoting healthy ageing. Previous work has assessed the prospects of digital technologies for health promotion and disease prevention in older adults. However, to our knowledge, ours is one of the first attempts to specifically address the use of wearables for healthy ageing, and to offer ethical insights for assessing the prospects of leveraging wearable devices in this context. We provide an analysis of the considerable opportunities associated with the use of wearables for healthy ageing, with a focus on the five domains of intrinsic capacity: locomotion, sensory functions, psychological aspects, cognition, and vitality. We then highlight current limitations and ethical challenges of such approach to healthy ageing, including issues related to access, inclusion, privacy, surveillance, autonomy, and regulation. We conclude by discussing the implications of our analysis in light of current debates on the ethics of digital health, and suggest measures to address the identified challenges.

## Introduction

**T**he COVID-19 pandemic has been a wake-up call to reconsider health priorities and modernize health systems, clearly showing that older adults are among the most vulnerable members of our communities. COVID-19-related mortality has predominantly been among those aged 65 and older, due to the concomitant presence of chronic conditions (1). At the same time, with reduced possibility for face-to-face interaction, the pandemic has led to a rapid increase in the use of digital technologies for healthcare, including monitoring the health of older adults (2, 3).

Taken together, the urgency to address health needs specific to rapidly ageing populations and the opportunity to leverage digital technology to modernize healthcare, invite a careful reflection on how to achieve healthy ageing objectives with the aid of digital health innovation.

In this paper, we consider a specific type of digital technology – wearable devices, i.e. electronic devices that can be worn directly on the body and are capable of sensing, storing, processing as well as sending and receiving data about the user over the Internet (4). We provide a detailed analysis of associated opportunities and challenges for using wearables to promote healthy ageing. Previous work has assessed the prospects and limitations of digital technologies for health promotion and disease prevention in older adults (5), but to our knowledge, the present paper is among the first attempts to specifically address the use of wearables for healthy ageing and to offer ethical insights for assessing their potential.

We begin by introducing the concept of healthy ageing. We then identify opportunities for wearables in the five domains of intrinsic capacity for healthy ageing: locomotion, sensory functions, psychological aspects, cognition, and vitality (6). Next we illustrate existing limitations and ethical challenges in relation to access, data quality, privacy, autonomy, and regulation. Finally, we discuss the overall findings in light of current debates about the ethics of technology and digital health, suggesting measures to address the identified challenges.

## Healthy Ageing and the Domains of Intrinsic Capacity

Healthy ageing is defined as “the process of developing and maintaining the functional ability that enables wellbeing in older age” (7). The healthy ageing paradigm suggests a shift in current models of care. Public health interventions are typically conceived to anticipate diagnosis, prevent disease onset, or delay progression, while clinical care models are centred around response to the clinical manifestation of disease (8). The paradigm of healthy ageing focuses instead on a variety of health determinants – behavioural and clinical, as well as social and environmental – that affect the progressive decline of individual functional capacity throughout the life course, across a broad spectrum of domains (9). From a population health perspective, the aim of this approach is not to increase average longevity but to improve health span, that is, to reduce morbidity across the spectrum of age-related chronic conditions that impair functioning in older adults.

Current research in the field of healthy aging at understanding which preventive and health-promoting interventions strengthen intrinsic capacity (10). Intrinsic capacity refers to the physical and mental characteristics that enable individuals to function, including health attributes allowing people to do the things they deem valuable and important. Five distinct domains of intrinsic capacity have been identified and validated: 1) locomotion and mobility functions, including balance and muscle strength; 2) sensory capacity and functions, such as vision and hearing; 3) psychological capacity, comprising mood and emotional vitality; 4) cognition, including subdomains such as intelligence and memory; and 5) vitality, measured in terms of metabolic and homeostatic capacity (6, 10).

Considering their specificity and significance for both current research and policy, in the following we use the five domains of instrinsic capacity as a way to capture the full breadth of the functional domains that matter to healthy ageing.

## Wearable Technologies for Healthy Ageing: Opportunities Across Domains

### Locomotion

In healthy ageing research, measurements of mobility (gait speed, for example) have been correlated with health-related outcomes such as life expectancy, inflammatory status, aerobic capacity, atherosclerotic formation, pathophysiological modifications, hospitalization, and falls (9). Wearable devices hold great potential for assessing individual levels of physical activity, as they are already used to monitor functional trajectories linked to mobility and to provide tailored feedback.

A host of commercially available wearables such as fitness bands and smartwatches enable monitoring of physical activity. These same tools could be employed to detect decline in locomotion or reduction in gait speed, for instance via validated mobility tests (11).

Muscle strength correlates with mobility, and thus with individual autonomy, for example in the execution of everyday tasks like climbing stairs. Wearable devices are already used to measure muscle strength via simple validated tests, such as the up-on-the-toes test (12). Muscle strength is also a protective factor against falls. Incidence of falls increases with age; approximately one third of adults over 64 suffer from a fall every year (13). Wearables are relatively inexpensive tools that can detect a fall and summon help (14). Like smartphones, most wearables feature accelerometers that can detect a fall, triggering an alert to caregivers or emergency services. Furthermore, such devices can estimate the risk of future falls, and offer targeted interventions to individual patient (15). Therefore, wearables such as smartwatches are increasingly used to detect falls but are also currently being explored for their abilities to predict and prevent falls in older adults (16).

### Sensory Functions

Sensory deficits such as vision and hearing impairments play a major role in determining how individuals function in their dwelling environment, maintain social relations, engage in physical activity, and preserve a sense of autonomy. Current uses of wearables go in the direction of preserving or improving sensory function for healthy ageing purposes.

Wearable technologies such as smart glasses have been studied as aids for people with sensory impairments such as colour blindness (17). Vibrotactile belts are used together with ultrasonic sensors and accelerometers to send vibrotactile stimulation to users with impaired vision, enabling them to avoid obstacles on their path (18).

Hearing loss is not only a functional impairment, but is also associated with impaired cognitive performance, and it is considered a risk factor for dementia and age-related psychosocial conditions such as depression, anxiety, reduced verbal communication, and social isolation (19). Hearing aids have been shown to significantly improve cognitive performance in older adults (20). Moreover, a recent study demonstrated that hearing aid users are less likely to develop mild cognitive impairment (21). These findings show that concrete uses and applications are already available and encourage further research and development for hearing aid technologies, to promote improved intrinsic capacity in multiple functional domains.

Such efforts can leverage recent progress in so-called ‘hearables’ (smart in-ear devices such as earbuds), and the merging of this technology with conventional hearing aids and cochlear implants (22). Connectivity enables seamless integration of ‘hearables’ with smartphone functions such as calls or messaging apps (thus improving social interaction) as well as provision of tailored recommendations based on an array of monitoring functions.Therefore, the availability of wireless connectivity in commercial and medical-grade hearables holds promise to further expand the use of hearing aids and implants.

### Psychological Functions

Mental health is an important aspect of healthy ageing, and wearables can help here as well. A healthy lifestyle can have a positive impact on mood, and can contribute to a purposeful outlook that can counterbalance senile depression, anxiety disorders, or apathetic tendencies that can develop in older adulthood (23). Wearables as well as other digital health technologies can monitor and passively assess subjective experiences, as for instance journaling apps allowing users to describe their mental health status throughout the day. In terms of active measurements, many wearable devices track physical activity and sleep to infer mental health status. For instance, wearable sensors can continuously collect data about physical activity (a correlate of mood-related issues (24)) and sleep time, including conditions related to sleep quality, as inferred by sleep patterns (25). Wearable-based virtual coaching systems can assist older adults in modifying health behaviours, and support them in finding a healthy balance between physical activity and rest (26). By fostering behavioural change in areas such as sleep, fitness, and lifestyle habits, wearables can promote emotional and psychological health (27).

Wearables can also enable the direct measurement of mood and cognition (28). Wearable electroencephalogram devices, for instance, can assess a user's mental state (29), and promote relaxation when used in combination with other tools such as virtual reality headsets and olfactory interfaces that affect information processing to modify mood and cognition (30).

### Cognition

Research examining the relationship between age-related decline in cognitive function and healthy ageing is ongoing. The notion of cognitive frailty, for instance, was originally introduced to describe the concomitant occurrence of frailty and cognitive impairment in the absence of neuropathological conditions such as dementia (31). Several studies are currently testing the ability of wearable devices to identify frail individuals at risk of cognitive impairment (32). Such studies are generally based on the correlation between mobility and sleep patterns, with cognitive performance measured through standard methods.

Patients with cognitive impairment and early signs of Alzheimer's disease commonly blink at a reduced rate (33). Wearable devices such as smart glasses that can monitor blink rate can thus infer clinical states related to psychological health and cognition (34). Similarly, other studies have observed the correlation between gait speed and cognitive frailty, concluding that gait speed deterioration as measured with a wearable device can indeed be associated with cognitive impairment in frail individuals. This suggests that gait speed measured by wearables can be considered a digital biomarker of cognitive frailty (35).

Finally, wearables can serve as warning and reminder tools for patients with early-stage cognitive impairment. Non-intrusive smart devices (in the form of clothing, shoes, jewellery, or watches) can vibrate or emit a sound to remind users to perform actions such as taking medication or brushing teeth, or to signal possible danger – for example, if an individual wanders from home without a mobile phone (26).

### Vitality

In the healthy ageing context, vitality is understood as the set of body functions dedicated to metabolism, or the transformation of food into the energy an organism needs. The balance between energy intake and expenditure constitutes an important factor in the ageing process, as energy expenditure tends to decline with age. For example, weight loss or low body mass index can be signs of malnutrition in older adults and can thus be targeted for interventions, to prevent what is known as the disabling cascade (36). Modifications in energy metabolism have been shown to be particularly influential for the ageing process (37), and healthy nutrition thus plays an essential role. For instance, having a balanced diet improves cardiorespiratory function and can help prevent common conditions of old age such as sarcopenia (38).

Wearable technologies can be used as a basis for inferring vitality measurements, although few concrete applications are available so far, such as in the case of tooth mounted sensors to detect food consumption (39). For example, necklace-like sensors can monitor eating habits by acquiring acustic inputs from the throat and also detect possible related diseases like dysphagia and indigestion (40). Most wearables track diet and food intake by relying on individual users to manually input their diet informations into apps, rather than directly monitoring calories consumptions (42).

Digital strategies and wearable tools can also support behavioural change related to maintaining healthy dietary patterns. Some of the most effective wearables in this area are currently been developed to track glycaemic levels in type 2 diabetes patients (43). Other aspects of vitality are linked to cardio-respiratory function, which can be monitored by wearables such as smart belts with embedded textile electrodes (41).

## Challenges and Ethical Considerations in the Use of Wearables for Healthy Ageing

### Access and Inclusion

According to recent figures, one fifth of the adult population of the US currently uses a wearable device. However, such figures change considerably when considering older users, who may lack the literacy, interest, or economic means to adopt new digital technologies like wearables and related devices such as smartphones that are often necessary to use them (44). This is reflected in the area of clinical research, where studies investigating digital health often require participants to bring their own device, resulting in demographic imbalances related to age, ethnicity, gender, socioeconomic status, and education background (45).

Partly as a consequence of marketing strategies, commercially available wearables rarely incorporate design features that address specific needs of older adults. Moreover, the proprietary nature of commercial wearable technologies may present a challenge for doctors or healthcare centres wishing to utilize simple validated tests, for instance in the domain of mobility.

Given the growing importance of digital technology in healthcare, some scholars argue that the ability to access and use digital technologies should itself be considered a social determinant of health (46). Barriers to access and lack of inclusive design for older users are thus concerning from an ethical point of view, since lack of access to digital technology can exacerbate existing health disparities that already place older adults at a disadvantage. In order for wearables to equitably serve as tools for healthy ageing, age-related digital gaps must thus be addressed (47).

### Data Quality and Representativeness

An additional challenge to the use of wearables for healthy ageing is the quality and validity of wearable data. If older adults generally have less access to or make less use of digital health technologies, more data comes from younger individuals, and the possibility to extrapolate medical information specific to older age groups is thus compromised (45, 48).

Moreover, since most health-related applications and wearable devices are created with young adult users in mind, and further developed through analysis of data coming from a younger population, older adults may be systematically underpresented and thus disadvantaged in terms of user experience and outcomes. From an ethical perspective, this may reflect an ageist prejudice foreclosing opportunities for older adults to benefit from digital health innovation.

Consider, for example, the Apple Heart Study, one of the largest attempts to date to use a wearable device (the Apple Watch) to analyse cardiac function in the general population (49). The sensitivity of the algorithm used for atrial fibrillation detection, based on cardiac data collected by an Apple Watch, drops from 96% to 50% when examining data from older cohorts (mean age 76 years old) in comparison to the official reports provided by the company (50). This is not just a technical matter of device accuracy, since the sensors may be very accurate in terms of what they track (e.g. heart rate). This issue can rather be traced back to data quality owing to the fact that data used to develop technologies such as sensors is not equally representative of different user groups, thus leading to underperformance, for instance, in older patients. Because it is uncertain that data collected through wearable technologies can be extrapolated to different age groups, greater efforts must go towards attenuating this type of demographic bias in datasets used to develop wearables heath technologies.

### Privacy and Surveillance

Wearables collect large quantities of personal data, with extensive and continuous collection arguably required to monitor multiple domains of intrinsic capacity in older adults over time. Such pervasive monitoring can be intrusive and pose risk to individual privacy, as identified in the literature (51, 52, 53). Surveillance today can sort people into categories, assigning worth or risk in ways that have real life consequences. Profound discrimination can occur, thus making surveillance not merely a matter of personal privacy, but also of social justice.

Device developers and actors involved in data collection, interpretation, or analysis have access to sensitive personal data that cannot be fully anonymized, due to the need to relay information back to users. This constraint increases the risk for unauthorized access or discriminatory use of personal data, as even seemingly trivial data (e.g. step count) can reveal sensitive information about users, such as location or health condition (54). Circulation of large volumes of individual data increases the risk for what has been termed ‘social sorting’ or surveillance practices that place people into categories, eventually leading to discrimination – a risk that is particularly serious for older adult already facing ageism (55).

Opaque data collection practices are not uncommon in the area of wearables, undermining informational self-determination, or the individual right to control information about oneself (56). As a consequence older adults may be vulnerable to intrusive practices, such as targeted advertising or use of data for fraudulent purposes (57). Adequate informed consent, data protection measures, and careful data oversight are key for clarifying how data will be collected and used, by whom, and for what purposes, as well as preventing risk of unauthorized and ethically dubious activities by actors such as big tech, health insurers, or governments. These elements are not new in the wider debate on the ethical issues of digital health, but present a higher degree of urgency when they involve specific user groups such as older adults (58).

### Individual Autonomy

The intrusive nature of wearables and digital technologies can be particularly problematic in relation to older adults. Due to potentially lower levels of digital competency, this segment of the population is less likely to be aware of risks specific to digital technologies. In particular, technologies that provide individualized feedback to users, like suggestions about health-promoting behaviours, may interfere with personal autonomy and self-determination. While nudging and its impact on autonomy are common features of public health campaigns and are not exclusive to digital technologies (59, 60), wearable technologies present specific ethical challenges because they are embedded in user's daily activities (61).

Wearables can urge users to modify their behaviour towards predefined health standards in exchange for rewards (e.g. gamified badges and prizes, lower health insurance premiums, social endorsement and acceptance). However, such standards do not necessarily take into account specific user needs, values, or measures of well-being. If wearables used to promote healthy ageing are direct-to-consumer, non-medical devices, the health standards utilized do not necessarily reflect validated measures or accepted public health standards, nor do such technologies undergo rigorous independent testing with adequate oversight. Technology developers and software architects may set standards in a way that encourages users to purchase products and services, driven by commercial rather than health-related purposes (62). For example, the use of wearables to screen for conditions such as arrhythmia has recently been criticized because of the high number of false positives, which in turn could lead to increased anxiety and distrust among users (63, 64).

Wearables can persuade users to undertake behaviour change without adequately engaging them in the process. Users can thus be influenced – nudged – in their decision-making, while remaining unaware of a technology's effect on their decisions and actions (51, 52). This invites careful ethical consideration of the risk that nudges from wearables could result in forms of more or less direct coercion.

### Efficacy and the Limits of Regulation

Wearable devices for healthy ageing include both medical-grade and direct-to-consumer tools. In both cases, tools may or may not be designed specifically to promote healthy ageing. As a consequence, crucial aspects may go unchecked, such as specific needs linekd to service continuity and the risks connected to extensive data collection and constant user monitoring.

Medical-grade wearables undergo regulatory oversight processes (e.g. clinical validation) that are not required for commercial devices such as fitness bands, unless they make an explicit health-related claim or provide medical information to users. This lack of regulatory oversight is problematic from an ethical perspective. Reasons for concern go beyond risks of physical harm, as monitoring wearables can be considered overall safe to use. It is when it comes to efficacy and quality that, absent adequate third-party controls, commercial devices may fail to meet accepted medical standards and user expectations.

Recent studies of patients using wearables to track their health after surgery have shown that wearables can vary greatly in terms of quality and accuracy, creating confusion, anxiety, and doubt in patients (65). This shows the need for more standardisation of what the devices can track and how they do so. In some domains of intrinsic capacity, as we have seen, further evidence is needed to determine whether wearables can be effective tools. For instance, clinical trials have shown that, while wearables can promote physical activity, it is still uncertain that they offer any advantage over traditional approaches to weight loss (66). Moreover, the efficacy of wearable-mediated interventions to promote healthy ageing, both at the individual and population level, is hard to measure. Ideally, the use of wearables to promote healthy ageing would be tested in large trials of adequate duration, with strategies to monitor functional decline and interventions to improve intrinsic capacity. However, lack of interoperability and limited data portability create barriers for data integration and comparison over different platforms (48). Specific ethical standards of clinical research should be in place, including decentralized trials, to ensure that such trials based on extensive collection of personal data are conducted in an ethically adequate way (67).

## Discussion

The uptake of a given health-related technology is not only a matter of how good, accurate, or efficient that technology is (68, 69, 70). Empirical studies provide a nuanced picture highlighting a variety of factors influencing technology acceptance on the part of older users. Education level and socioeconomic status have been shown to positively correlate with propensity to use smart wearables for health monitoring (71). Perceived functional value is a driver of continued intention to use smart technologies by older users (72). But interestingly, also a product's ability to arouse curiosity, to satisfy users' desire for knowledge, and to influence emotions in a positive way play a crucial role in determining adoption over longer periods of time; whereas inertia and technology anxiety represent clear impediments (72). Self-perception linked to wearing techological artifacts is also important. For some older users, wearable healthcare technonlogies can be problematic as they carry an image of being unhealthy; other users, however, are positively influenced by a technology's ability to improve life's quality or to make one's life more convenient (73). User acceptance, however, is not a fixed variable. Studies show that, quite to the contrary, it can be shaped through appropriate communication and user training, and that user friendly interfaces designed to address the needs of older users can greatly influence propensity to use (74).

Based on the above considerations, specific attention should be paid to the social conditions that enable or impede successful use, including the ethical aspects we have identified and discussed. For instance, aspects such as usability and intrusiveness are likely to represent major obstacles to the uptake of wearable solutions, especially for users who may be less at ease with digital technologies in the first place. Independently of how advanced or accurate the device is from a technical point of view, we should not think about these technologies as existing in a vacumm – if they are to become tools for our ever-ageing population, attention should be paid to the contextual requirements and specificifities of these users. In this direction, we welcome proposals that move away from conventional approaches to medical device validation and go towards validation frameworks that test and validate devices in concrete environments and against concrete, age-specific clinical and functional outcomes (75). Involving specialists such as geriatricians, health policy experts, and end users in the design and testing of wearables for healthy ageing purposes is key to realizing their potential.

The need to consider the social conditions that enable or impede successful uses of wearables for healthy ageing calls for integrating wearable technologies with health services that offer advice and proven interventions. A notable example that goes in this direction is the digital health suite developed under the aegis of WHO, in the context of the iCOPE (Integrated Care for Older People) program. The iCOPE MONITOR app enables the continuous collection and analysis of real-world ecological health data, monitoring all domains of intrinsic capacity. When the app detects deterioration in any domain, it activates a response by qualified personnel. iCOPE MONITOR was successfully implemented in the Occitania region in France, in a population of about 200,000 individuals aged 60 and above (59). While the iCOPE initiative does not yet incorporate the use of wearables, it illustrates the potential of dedicated digital health monitoring and digital health promotion in the domain of healthy ageing. Success in integrating wearable technologies into such programs depends significantly on validated digital biomarkers that capture fluctuations in intrinsic capacity in older adults (60). Digital technologies need to be embedded in a system in which health care professionals and social workers can be quickly notified when signs of deterioration occur, for the technology to provide tangible benefit.

Besides addressing individual needs, such systems enable analysis of how specific deficits can affect older adults from different socioeconomic backgrounds differently, and how interventions might address systemic factors of deterioration in intrinsic capacity in a more equitable way. Most likely, wearable technologies will prove effective for promoting healthy ageing not merely as individual devices, but as part of a broader socio-technical ecosystem, in which monitoring practices are connected to clinical services operating in a concerted fashion to meet the specific needs of older adults. In this way, differences in functional trajectories that are due to systemic factors such as socioeconomic determinants of health are more likely to be addressed in equitable and efficient ways. Consider also the attention devoted to equity and fairness in the domain of digital health (48, 63, 64, 76, 77). These issues are particularly relevant in the face of structural injustice in the form of ageism, racism, social inequality, and poor education. Ethical frameworks addressing the structural aspects of health inequality have long been discussed (78), but special attention should be paid to ageist prejudices present in contemporary societies, including in the domain of healthcare and public health (79). Social structures leading to systematic age-based exclusion and inequality need to be analysed and addressed also in relation to the role that digital technologies play in perpetuating those structures.

Moreover, the hetherogeneity of the five different domains of intrinsic capacity must be taken into account. Wearables are particularly well suited for tracking physical activity, but less effective in other domains of intrinsic capacity such as vitality. Similarly, functional decline may be more effectively measured in some domains of intrinsic capacity than others. For example, current measures of cognitive decline (e.g., Mini Mental State Examination, MMSE) are likely to detect functional impediments too advanced to be addressed or slowed down, lessening the utility of wearables for the promotion of healthy ageing in the domain of cognition. Novel biomarkers should therefore be developed and validated to enable the detection of early signs of cognitive deterioration, and trigger interventions that delay or prevent the onset of progressive conditions such as dementia (19).

Our analysis warns against simple and futuristic tales that describe technologies such as wearables as ready-made solutions to the challenges of ageing populations. Yet, current discussions on digital health for aging populations often paint a future where digital technology is extended to more and more aspects of the lives of older adults, as various smart sensors on the body and in the home monitor health conditions and constantly transmit data to cloud servers (5). In addition, as we have seen, digital technologies are often not tailored to the specific needs and requirements of older adults.

As we have seen so far, several challenges loom large on the use of wearables for healthy aging. Most of these challenges cannot be easily addressed by technological fixes alone, as in the case of issues related to access and inequality. More generally, healthy aging strategies pertain to the health and well-being of older individuals representing a growing fraction of the overall population. It is therefore crucial to recognize that such choices involve matters of public health and public policy rather than being solely confined to technological developments.

Digital technologies are frequently presented as inherently flexible artifacts that developers and users can shape, reconfigure, and transform according to their specific needs and interests. In fact such characteristics are not present a priori, as technical systems often come with specific characteristics that can prevent them from being interpreted flexibly and, for instance, being adapted for health-related purposes (80). In the context of healthy ageing, wearables may need to enable features not included in their initial design – especially if we consider commercial tools such as fitness bands. Considerable coordination among stakeholders (e.g. developers, researchers, oversight bodies, and users) is thus required to re-interpret a device's original design and use purpose to enable monitoring and preservation of functional ability and active engagement throughout a person's life (81, 82).

## Conclusion

Wearables are currently the focus of significant interest, including their prospects to innovate the health sector. Our analysis has highlighted several opportunities for the use of wearables to promote healthy ageing. We also illustrate critical impediments and ethical concerns that must be addressed to fully exploit the opportunities offered by such technologies for healthy ageing. In light of our analysis, we suggest that wearable technologies for healthy ageing should not be understood as mere individual gadgets, but should instead be embedded in a broader health-promoting ecosystem in which healthcare professionals and social workers operate in coordination to respond to the evolving needs of older adults.

*Authors' contribution:* All authors contributed equally to the conceptualization, data collection, analysis, interpretation, drafting and writing. All authors approved the submitted version of the paper.

*Funding note:* Open access funding provided by Swiss Federal Institute of Technology Zurich.

*Declaration of interest:* the authors declare no competing interests.

*Ethical Standards:* n/a.
